# From pandemic to podium? Norwegian Olympic handball players’ journey to Tokyo 2020

**DOI:** 10.3389/fpsyg.2025.1566238

**Published:** 2025-06-05

**Authors:** Eline Aase, Frank Eirik Abrahamsen, Henrik Gustafsson

**Affiliations:** ^1^Department of Sport and Social Sciences, Norwegian School of Sport Sciences, Oslo, Norway; ^2^National Research Center for Youth Sport Studies, Department of Educational Studies, Karlstad University, Karlstad, Sweden

**Keywords:** elite athletes, performance, stress, psychological preparation, coping, COVID-19, Olympic Games

## Abstract

**Introduction:**

Mental preparation ahead of the Olympic Games (OGs) has been an area of interest for sports psychology researchers over several decades. However, there are few studies based on athlete perspectives of their experiences coping with pressure at this competition level. The COVID-19 pandemic also placed athletes in a demanding situation as they had to deal with the suspension of sport activity, isolation, and general uncertainty—culminating in the first postponement of the OGs in peacetime. Athletes had to balance coping with everyday life in a pandemic with navigating training in ever-changing conditions, indicating it was particularly valuable to investigate mental preparations ahead of the Tokyo OGs.

**Objective:**

The current study aimed to explore how Norwegian handball players of various experience levels mentally prepared for the Tokyo OGs and how they experienced their preparations during the COVID-19 pandemic.

**Methods:**

Retrospective semi-structured interviews were conducted with seven handball players (four women, three men) who participated in the Tokyo OGs. A reflexive thematic analysis was completed to examine the findings.

**Results:**

The findings are described in two overarching themes: (1) failing to plan is planning to fail, and (2) balancing life and sports in a pandemic. Extensive preparations were done on an individual and team level. These incorporated mental, tactical, physical, and practical elements. Individual efforts varied and there were indications of certain team differences. The pandemic made the players’ everyday lives unpredictable, which was mentally exhausting for some. They coped with the uncertainties in different ways, though this often entailed focusing on the positive aspects. Overall, the players’ respective contexts affected their perceptions of the pandemic and the postponement of the OGs, and their appraisals of various stressors and subsequent coping strategies.

**Conclusion:**

The indications of team differences and variations in individual preparations imply that there was no “correct” way to prepare—all roads led to Tokyo. Experience was beneficial in several ways, including coping with the Olympic environment. Some found coping with the effects of the pandemic mentally exhausting, thus potentially affecting preparations. Still, the players got to practice dealing with unexpected events, which could aid future coping efforts.

## 1 Introduction

Attaining peak performance at the Olympic Games (OGs) is a multifaceted challenge. It begins with the strenuous journey to qualify, which often takes dedicated athletes decades. Once on the national team, the struggle shifts to retaining a roster spot. As the Games approach, the escalating pressure to excel becomes inescapable. Norwegian national team handball player Endre encapsulates the daily strain elite athletes face:

“That’s the most challenging thing, then, as an athlete—it’s not the number of training hours or traveling or anything—it’s the pressure that’s hardest. That’s what people do not understand: How demanding it is to have exams twice a week where all eyes are on you.”

Consider the OGs, then—the zenith of an athlete’s career, occurring just once every 4 years and subject to intense global scrutiny, where every match is akin to a high-stakes examination. How might athletes steel themselves against the colossal stress inherent to the OGs, particularly in the face of a worldwide pandemic? Gould and Maynard (2009) indicated in their review that studies on Olympic athletes’ coping mechanisms span various approaches, and they advocate developing a range of strategies. They also argued that current research addressing the psychological development of OG athletes is insufficient. Athletes, coaches, and researchers must comprehend the psychological and physical factors at play for optimal performance in high-pressure moments.

Postponed due to the COVID-19 pandemic, the 2020 Tokyo OGs were ultimately held in 2021. This study explored the profound psychological stress and resilient coping strategies of Norwegian handball players, who confronted unparalleled challenges in preparing for these rescheduled Games amidst the pandemic. Reflecting literature on Olympic pressure, choking, mental preparation, and the nuances of Norwegian sports culture (Hansen et al., 2021; Skille et al., 2020), the article also considers pandemic influences on Olympic readiness, referencing Lazarus (1966) and Lazarus and Folkman (1984), and sports coping studies.

Performance anxiety can undermine elite performance. While some athletes excel under pressure, others may falter or ‘choke.’ Various studies have examined factors leading to underperformance and choking in sports (e.g., Beilock and Gray, 2007)—including soccer penalty shootouts (Jordet et al., 2009) and basketball free throws (Maher et al., 2020). There are numerous theories explaining how stress and anxiety impact performance and choking, but they are primarily based on two attention-based theories: self-focus and distraction theories (for a review, see Gröpel and Mesagno, 2019). While not explicitly part of the current study, these theories are crucial in guiding elite teams to mitigate stress and anxiety during the OGs.

Self-focus and distraction-based interventions aim to maintain task-relevant focus while reducing conscious skill control (Gröpel and Mesagno, 2019). Research indicates that athletes can lessen the risk of skill failure under pressure by learning to manage attentional demands (e.g., Beilock and Carr, 2001). Methods like acclimatization, simulation training, and dress rehearsals help athletes familiarize themselves with performance stimuli and thereby reduce distractions and boost performance (see, for instance, Mesagno et al., 2008; Mesagno et al., 2015).

Acclimatization interventions have shown effectiveness in coping efforts (Hill et al., 2011; Oudejans and Pijpers, 2009, 2010), and one recommendation is ‘overtraining’ for worst-case scenarios (Williams et al., 2015). Recent findings, such as from Low et al.’s (2021) meta-analysis, support the effectiveness of pressure/acclimatization training, advising the increase of pressure during training. This may be crucial for events like the OGs, where pressure is elevated and everything becomes a performance issue (McCann, 2008).

This study explores Norwegian handball players’ strategies to navigate their OG preparations amidst the Tokyo OGs’ postponement challenges. Sports psychology consultants (SPCs) highlight the unique prestige of the OGs as the apex of athletic achievement (e.g., Arnold and Sarkar, 2015; McCann, 2008). The Games present unique stressors such as selection pressures, performance expectations, distractions, and future planning (Arnold and Sarkar, 2015). OGs are considered defining moments for athletes, distinct from other competitions (McCann, 2008). Pensgaard and Duda (2003) liken the OGs to a “competition with a life of its own,” underscoring their unique challenges and prestige.

Past research has examined successful team preparations for OGs without epidemics. For instance, successful teams in the Atlanta OGs employed substantial mental preparations, with performance success associated with well-developed competitive plans and routines (Gould et al., 1999). Gould and Maynard (2009) reviewed research on psychological preparations for Olympic performance and emphasized the long-term nature of Olympians’ development—encompassing both athletic and psychological aspects. Recounted preparations for Olympic success involved planning (e.g., distraction readiness, plan adherence), physical training (e.g., managing training load, international competition participation), mental training and sports psychology (e.g., readiness for unforeseen events, integration of mental training), team elements (e.g., confidence, cohesion, coaching, support personnel, team training, selections), and external factors (family/friends, media/sponsors, equipment/travel, environmental conditions).

However, little is known about how the extra toll of a pandemic and subsequent postponement affected athletes’ OG preparations—with two recent investigations being exceptions. Rogers and Werthner (2023) explored Canadian athletes’ experiences of preparing for the Tokyo OGs. They discovered that the athletes used the pandemic as an opportunity to reflect on life in and outside of sports, and their circumstances (e.g., injury status, stage of career, first or last OGs) influenced their perceptions of the pandemic and the postponement of the OGs. Oblinger-Peters and Krenn (2020) reported that the postponement had varied effects on participants. Three main themes emerged: immediate emotional reactions (confusion, disappointment, relief), associated consequences (prolonged pressure, decreased motivation, performance concerns, but also improvement opportunities), and coping strategies (distancing from sports, reframing, seeking acceptance, planning).

The 2020 Tokyo OG were unprecedented—rescheduled from 2020 to 23 July to 8 August 2021 due to the COVID-19 pandemic. The delay, necessitated by public health concerns and logistical challenges, disrupted athletes’ intricate plans for training, nutrition, and competition (Oblinger-Peters and Krenn, 2020). The postponement introduced additional challenges, including maintaining peak physical fitness over an extended period, managing psychological stress and potential economic concerns, and adapting to logistical hurdles such as venue access, travel restrictions, and rescheduled international competitions. Likewise, research has explored various contexts involving elite athletes, including the effects of the COVID-19 pandemic (see Lundqvist et al., 2022; Pedersen et al., 2021; Urbański et al., 2023). These studies show how elite sports struggled to cope with the pandemic and how to reopen for elite sports competitions. Urbański et al. studied elite athletes with disabilities preparing for national and international sports events. They found that athletes’ mental health and training opportunities were at increased risk during the pandemic, whereas individual coping styles were inadequate to handle the stress. Pedersen and colleagues’ study was male elite soccer players. They suggested that controlled reopening with strict protocols could be safe, although this entailed a strict protocol with surveillance and compliance with guidelines from players and the clubs. In the professional statement paper, Lundqvist et al. (2022) provide an overview of the psychological challenges reported by different countries regarding the Tokyo OG, including topics such as stress and anxiety, loss of routine, and life-balance challenges.

Under similar circumstances, the Norwegian male and female handball teams adapted to the new reality. The Norwegian goal of eight medals (all sports) at the Tokyo OGs was realized. The male team exited in the quarterfinals, while the female team clinched a bronze medal despite pre-game predictions favoring them for gold. While altered preparations and high expectations can influence performance, securing a bronze in the OGs remains a significant achievement. How the Norwegian teams navigated the stressful year leading up to and the Tokyo OGs themselves during the pandemic may provide valuable insights for future calamities.

Gould and Maynard (2009) underscored the necessity for theoretically driven studies. We, therefore, draw from the extensively utilized framework of Lazarus and Folkman (e.g., Lazarus, 1966; Lazarus and Folkman, 1984)—also used in elite sports (e.g., Pensgaard and Duda, 2003; Uphill and Jones, 2007). Their theory emphasizes the dynamic relationship between individuals and their environment and suggests that stress responses arise when a situation exceeds an individual’s resources, an imbalance potentially affecting their well-being (Lazarus and Folkman, 1984). This framework accounts for conscious and unconscious factors during the appraisal process (Lazarus, 1999). Lazarus and Folkman (1984) explained that we evaluate potential outcomes and coping options in primary and secondary appraisals.

Primary appraisals categorize encounters as irrelevant, benign-positive, or stressful, with three subcategories within the stressful category—harm/loss, threat, and challenge (Lazarus and Folkman, 1984, 1987). The pandemic, for instance, could make OG preparations stressful and pose a threat to participation and performance. Secondary appraisals, in response, assess possible actions and the degree of control over such outcomes (Lazarus and Folkman, 1984, 1987). In the context of the pandemic, these could entail strategies to mitigate its impact. Lazarus and Folkman (1987) also identified problem-focused and emotion-focused coping strategies, while avoidance, another coping strategy, offers temporary relief but is often ineffective (Madigan et al., 2020).

We grounded this study in the perspectives of Lazarus and Folkman while contextualizing the findings within broader research. Novel challenges and drawbacks (e.g., Thatcher and Day, 2008), such as the pandemic, may present extra worries—even for experienced Olympians. The varying COVID-19 restrictions worldwide highlighted the potential inadequacy of preparations, conceivably intensified by athletes’ circumstances. Therefore, we took the opportunity to understand the distinctive situation of a pandemic during the most critical competition in the careers of many elite athletes. Considering the crucial and timely demand to comprehend the mental rigors elite athletes endure in preparing for the OGs, particularly amid the unprecedented challenges of a global pandemic, we posed the following essential research questions: How did Norwegian handball players of various experience levels mentally prepare to perform at the OGs in Tokyo? And how did they experience their preparations amid the COVID-19 pandemic?

## 2 Materials and methods

Below, we outline the methodology of the study, including theoretical assumptions, the researcher’s role, participant details, data collection and analysis methods, ethical challenges, and trustworthiness.

### 2.1 Theoretical assumption and disclosure of interests

This study adopted a relativist ontology and constructionist epistemology in conducting retrospective semi-structured interviews (Braun and Clarke, 2013). EA conducted and completed pilot interviews to account for her inexperience with qualitative research. HG and FEA, experienced in qualitative research and as SPCs (also in OGs), guided throughout the process. FEA, primary advisor and decade-long collaborator with the national handball federation, has previously published work on elite handball (e.g., Abrahamsen and Pensgaard, 2012; Skille et al., 2020). Conflicts of interest were nevertheless absent in the present study. EA’s sports psychology background aligned with the research process. All authors share a deep interest in elite performance and applied sport psychology.

### 2.2 Participants

Recruiting the Olympians posed a challenge. Access to elite participants requires creativity, thorough preparation, appropriate credentials, contacts, and a degree of luck (Odendahl and Shaw, 2001). We used purposeful sampling (Patton, 2002), with EA reaching out to potential participants. Out of eleven contacted, eight initially agreed, but one later declined due to time constraints. Consequently, seven handball players (four women and three men) participated. They averaged 10 years of senior national team experience (*M* = 9.7, SD = 6.0) and nine senior championships (*M* = 9.3, SD = 5.1) (Table 1).

**Table 1:** Participants and experience.

Interviewee/Pseudonym	Level of experience
Anna	First-time Olympian
Bea	Olympic experience
Caroline	First-time Olympian
Dina	Olympic experience
Endre	First-time Olympian
Fredrik	First-time Olympian
Georg	First-time Olympian

The women’s team has a long history of success in the European Championships (ECs), World Championships (WCs), and OGs. The men’s team has progressively improved and become competitive for medals (e.g., Håndballforbundet, n.d.). Yet they had not qualified for OGs since 1972, leaving most players and staff without Olympic experience. The sample size was mainly pragmatic due to players’ professional commitments across different countries. We used reflexive thematic analysis to analyze the data (Braun and Clarke, 2021b). The number of participants might be considered low. However, given the participants’ high level and experience, they brought rich insights pertinent to the study’s aim. In addition, with the narrow and focused aim for the study we considered the interviews providing sufficient information power, despite the smaller sample size (Malterud et al., 2015).

### 2.3 Procedures

EA introduced the project to players via team staff 3 months post-OGs. Details, emphasizing purpose, confidentiality, and gatekeeper endorsement, were shared. While face-to-face interviews were initially planned, all but one was held digitally due to COVID-19 restrictions and player availability. Despite potential communication quality concerns (Purdy, 2014), previous research has indicated participants’ preference for digital interviews (Archibald et al., 2019, pp. 3–4). Conducted from November 2021 to January 2022, the interview scheduling, chosen by players, avoided coinciding with championship preparations.

The interview guide followed DiCicco-Bloom and Crabtree’s (2006) guidelines ensuring a comfortable environment, with pilot feedback suggesting follow-up questions for initial questions found challenging. The guide served as a safety net that avoided leading athletes while providing probes for unexhausted vital points, in line with Sparkes and Smith’s (2014, p. 92) assertion that less frequent questioning enhances data quality.

Interviews were conducted in Norwegian and ranged from 64 to 94 min (*M* = 74, SD = 9.7). They were recorded on a Dictaphone before being transcribed verbatim (totaling 200 pages), translated to English, and were analyzed in MAXQDA. Another person listened to difficult-to-hear passages to ensure quality control during transcription (as mentioned in the consent form). These segments did not include personally identifiable information.

### 2.4 Data analyses

We completed a reflexive thematic analysis (e.g., Braun and Clarke, 2006, 2019), heeding the words of Braun and Clarke (2019) that qualitative research is about interpreting and creating context-bound narratives of meaning-making—not uncovering the “truth.” First, during data collection and transcription, EA familiarized herself with the data and made margin notes. Then, EA was supervised when continuing the analysis in MAXQDA. During the coding phase, EA highlighted engaging portions related to the research questions, naming them and grouping similar content. We aimed to capture players’ nuanced descriptions and to preserve narrative diversity (Braun and Clarke, 2006, p. 89). Discussion with others (i.e., supervisors, fellow students, and support personnel working with the teams) shaped thematic decisions, but player privacy was paramount in these dialogs. All researchers approved the final themes and quotes, and the final thematic map mirrors the meanings of the entire dataset (Braun and Clarke, 2006).

### 2.5 Ethics and trustworthiness

The Norwegian Centre for Research Data (Ref. nr. 275873) approved the project, and we had written informed consent from all participants. The players and teams have a public profile with potential identification risks. Therefore, selected quotes do not provide much identifiable information. We also presented the selected quotes to a person with extensive handball knowledge. This person could not identify the players.

Braun and Clarke (2006, 2019, 2021a) proposed several quality criteria for thematic analyses, such as specifying the thematic analysis type, being active in the research process and thorough when coding, giving equal attention to each data item, and balancing analytical narratives and extracts. Member reflections are often recommended (e.g., Braun and Clarke, 2013) but were deemed difficult after recruitment, as the players must have accepted more than one interview. EA actively reflected throughout the entire research process (reflexivity; Braun and Clarke, 2013) and kept a research journal (Sparkes and Smith, 2014) with intermittent entries noting how the interviews went (e.g., general tone/mood, rapport-building, body language) and points of interest. HG and FEA acted as critical friends (Smith and McGannon, 2018), offering alternative perspectives and promoting reflection by challenging explanations and interpretations.

As non-native English speakers, this document underwent language proofing (including with AI tools, namely ChatGPT and Grammarly) while adhering to usage guidelines and ethical standards and not creating new content. Importantly, no personal or sensitive data were relayed to these AI tools; thus, direct quotes have been retained with minimal alterations.

## 3 Results and discussion

This study examined how Norwegian handball players mentally prepared for the Tokyo OGs and how they experienced their preparations during the COVID-19 pandemic. The findings are organized into two descriptive themes. The first theme; Failing to plan is planning to fail, include the subthemes, Personal and team performance, the role of others and the significance of experience balancing life and sports in a pandemic. The second theme, Balancing life and sports in a pandemic, include the subthemes, How the pandemic affected the players’ lives and routines, uncertainty during the pandemic Olympic Games and rebounding from setback: post-Olympic reflections (see Table 2).

**Table 2:** Themes and sub-themes.

Theme	Sub-themes
Failing to plan is planning to fail	Personal and Team Preparations, The Role of Others, The Significance of Experience
Balancing life and sports in a pandemic	How the Pandemic Affected the Players’ Lives and Routines, Uncertainty During the Pandemic Olympic Games, Rebounding from Setback: Post-Olympic Reflections

### 3.1 Failing to plan is planning to fail

#### 3.1.1 Personal and team preparations

The theme failing to plan is planning to fail describes how the teams prepared collectively and individually. Preparations for the OGs encompassed mental, tactical, and physical training, nutrition, acclimatization, and practical considerations (e.g., COVID-19 guidelines and media and advertising regulations). The players opined that individual athletes can focus their training exclusively on the OGs, but in handball, there are essential games in their leagues, too. Anna said, “We have important things happening all the time.” She detailed their two-fold preparations—a training camp for team and game development preceding the final selection and a pre-camp in Japan for fine-tuning performance. The female athletes notably emphasized the importance of collaborative endeavors, such as establishing goal-oriented strategies before championships, wherein they discussed their ambitions and potential setbacks. Caroline emphasized team- and confidence-building activities but expressed that most mental preparations are individual.

As a result, the athletes highlighted various techniques incorporated in their preparations. For example, Endre acknowledged that his role diverged from his initial expectations and that he used goal-setting as a component of his coping strategy. Caroline visualized her success in pivotal matches and crafted mental diagrams alongside her SPC, detailing her strengths and weaknesses for use in her mental imagery. Similarly, Fredrik emphasized visualization in his OG preparation, while Georg and Anna routinely used it to anticipate various situations. Numerous players, like Fredrik and Endre, consulted their SPC regarding practical challenges:

“We’d been through a process of what the OGs were and how it worked and which pitfalls there were and stuff like that … everything from bringing your own pillow to being able to bring [Norwegian candy], to trying to find routines even in such a unique situation, everything really. So, I must say we have also thoroughly reviewed the mental aspect” (Endre) (Table 2).

#### 3.1.2 The role of others

All but one player worked individually with SPCs. The female players contacted SPCs through the National Olympic Training Center [NOTC], whereas the male players used external consultants and explained that they valued the objective source. After years of collaboration with her SPC, particularly with the national team, Bea emphasized the importance of acquiring knowledge and tools she could apply independently. In contrast, Dina contacted her SPC a year and a half before the OGs. While it positively influenced her everyday handball life, she hoped the benefits would be more pronounced during the OGs:

“The OGs were one of the main reasons I went in and tried to, yeah, get a more extensive collaboration with [SPC] a year and a half before to try somehow to work some things out. So, I feel like I really tried to prepare, but I do not quite feel that it helped, and therefore, when I look back on it, I do not really know what I should’ve done differently either because I truly tried.”

Norwegian Olympic Personnel (NOP, e.g., nutritionists, doctors, physiotherapists) were generally deemed necessary in players’ preparations. However, some players observed individual variances in athlete collaboration with NOP, noting that handball might not be the most engaged sport. The female team seemed more involved with NOP, as evident in Caroline’s statement about a physical trainer:

“It’s really fun because it’s directed towards handball. [She/he] has also taught the coaching team a lot more about what kind of quantity and what kind of physical training you should have, so I think that may be the most important part from [NOTC] that has come into our team.”

When prompted about simulation training, the female players mentioned training matches and tactical focus points (e.g., practicing the final game minute) and revealed that the team got up at 5:00 a.m. during a training camp to check their response to early game preparations. Bea explained that preparations for the OGs always differ depending on the location—predominantly concerning time zones and acclimatization. Acclimatization and nutrition were crucial focus points for many ahead of the Tokyo OG (e.g., Eijsvogels et al., 2021). Several players in the current study mentioned this, and both teams went through the necessary preparations. In particular, the men’s team had joint meetings covering the main nutritional challenges, whereas the women’s team had individual conversations with a nutritionist advising on how to sustain energy (e.g., using energy gel).

Both preparations over time and game preparations were tailored individually depending on preferences and needs. For the teams’ game preparations, meetings with video analysis were essential for assessing the opposing team’s playing style and tactical elements, such as determining how aggressive their defense should be. This way, everyone had a shared understanding of the game plan and their responsibilities. The latter corresponds with recommendations in team mental model research (see Filho and Tenenbaum, 2020).

Video was also used individually, though in distinctive manners. While Endre watched clips and at least one full game to acquire an overview of what characterized their opponents, Bea felt it could hamper her performance if tactical plans became too controlled. Anna adjusted her preparations depending on whether she was part of the starting lineup, while Fredrik emphasized following the same plan the day before and on game day (though noting that this becomes slightly different with the national team). Endre illustrated how the OG’s playing schedule (playing every other day) fluctuates from terrific to terrible days. He described moving his preparations into the game days:

“Game days are shitty. You walk around constantly thinking about handball, and you have this inner turmoil from you get up in the morning … to when you cannot sleep after you have played a game in the evening, so game days are simply shitty, it’s been that way my entire career. So, I often move, unless we have an early game … I move my preparations into that day, just to have something to do. I could have done it the day before but that day is so nice compared to the game day again, so I’d rather have one nice day and one day when you are truly at work.”

#### 3.1.3 The significance of experience

The players’ proficiency in dealing with challenges evolved positively over time—moving from large emotional fluctuations and fixating on errors to improved coping and focus on controllable aspects. Some improved confidence, others self-talk, and some became calmer. Two seasoned players noted significantly improved emotional balance, expressing joy in success and resilience in disappointment. Anna, a first-timer in the OGs, offered insights into the benefits of being less experienced:

“It’s always nice to sort of have as much experience as possible, but for my sake I also feel that when you have not been a part of that much you are also able to face it with a little bit of fearlessness in a way.”

The experience seemed to have improved the players’ ability to focus on the next opportunity instead of on their nerves. Nervousness was also considered a good thing, with Dina proposing that sometimes, “Feeling good is a bit overrated.” Still, nerves can be overpowering, and some advised that finding tasks and details to focus on could help avoid consequence-thinking. These findings align with previous research proposing that elite athletes interpret their anxiety as more facilitative compared with lower-level athletes (e.g., Hanton et al., 2008). A strategy mentioned by Georg and Fredrik was resetting—here, in Fredrik’s words:

“I also think it’s quite nice to sort of think about when you watch the game afterward that it was here, from this point I reset, and then you see that okay, from this point it went well and sort of learn that you can do it. Stop, and then say, “Okay, I’m resetting now.”

#### 3.1.4 Failing to plan is planning to fail—recapitulation

The preparation methods were in line with existing research on psychological readiness for the OGs, which includes physical, social, and situational factors (Gould and Maynard, 2009). Team preparations were organized based on time frames and divided into team-based and individual efforts. Team foci included goal-setting, tactical aspects, unity, and confidence-building—aligning with successful OG team research in the past (Gould and Maynard, 2009). The team coaches involved players in decision-making, emphasizing robust team value systems. The women’s team showcased a history of success and a culture of mutual support, while the men’s team, still developing, implemented actions for confidence-building (Hemmestad and Jones, 2019; Skille et al., 2020)—which was relatively evident from the present interviews.

Evolving research has explored training load, SPCs’ experiences, sports psychology interventions, and pandemic-era preparations (Arnold and Sarkar, 2015; Carr et al., 2022; Kvorning et al., 2017; Morris et al., 2022). Both teams engaged minimally in simulation training, rather relying on club experience. This finding is somewhat surprising. With added pressure and novelties, the OG environment poses challenges, particularly for first-time participants. Acclimatization, pressure training, and coping strategies can mitigate performance decrements induced by new stimuli, emphasizing the importance of familiarization (Beilock and Gray, 2007; Low et al., 2021). The present players seemed familiar with coping with typical club-playing situations, given the frequency and duration of their seasons. Additionally, annual championships offer ample opportunities to acclimate to the general championship environment—which may help explain the infrequent use of simulation training.

### 3.2 Balancing life and sports in a pandemic

The theme balancing life and sports in a pandemic summarizes how the COVID-19 pandemic altered the players’ daily lives and handball routines and how they experienced the uncertainties surrounding the postponement of the OGs and the OG experience.

#### 3.2.1 How the pandemic affected the players’ lives and routines

Players dealt with an unpredictable routine, including sudden game postponements and potential quarantines. The already long handball season was extended from August/September to May due to game postponements. Combined with the OGs during the summer, this created a two-in-one season that resulted in a significantly increased workload. This was mentally taxing for some and elicited varied coping responses. While Dina did not feel added stress and accepted that updates would arrive in due course, Fredrik noted instances of passive attitudes during game preparations, including his thinking, “It’s not certain that we’ll play anyway.” He portrayed a challenging setting:

“We may have been in the bus for 6 hours and then we have gotten an answer on the COVID tests that there is one positive, or two positives, and then we had to turn around and go home and spend 14 days in quarantine. So you went through a lot of mental preparations. You analyzed the players, got ready to play a game, you tried to get in the right mindset, and then it was just gone without you getting an outlet for it. So you often felt that you did a lot of preparations for nothing.”

The pandemic impacted the players both on and off the court. Handball activity ceased during the first lockdown, and a surge of serious injuries arose at the start of the new season. Georg encapsulated this concern, stating, “There was a period when you thought, ‘Okay, who will get injured now—this week.’” Outside sports, the pandemic led to less social interaction. Though the first lockdown afforded some players more home time, the following season resulted in prolonged periods without seeing friends and family:

“I live in [country] because I’m a handball player but it’s like, now that’s the only thing I could do and I have not, like I did not see [my family] for a year and a half, and that’s a very long time not to see those closest to you. I think that can sort of have an impact prior to a big championship also, because it’s always very specific beforehand anyways but it’s been very closed ahead of these OGs.” (Dina).

To cope with the uncertainty of the situation, Fredrik emphasized the importance of being social with teammates, both physically and virtually. He accentuated their focus on the positives, a common coping strategy for these players. For example, the first lockdown provided free time that could be used to implement more physical training and rest than usual. For the men’s team, this was considered an opportunity to gain an advantage:

“We can use this time extremely well and train very well, while others might not have the opportunity to do so. So, we tried to see it as an advantage, then, that when we get out of this we are gonna be even better trained than we were. And I think that most of the guys on the national team got a small boost from [that]—when they shut down and we agreed that, “Okay, we are gonna be the fittest team when we are done” (Georg).

The players also managed to accept the ambiguous situation surrounding the completion of the OGs in 2020. Maintaining perspective on the situation, some mentioned that varying restrictions would cause unfair competition. Still, Bea observed that her attitude might have been different had this been her first OGs, saying, “I know there were many who were very stressed and scared … and were very disappointed when it got canceled in 2020.” Nonetheless, there were substantial individual variations in players’ perceptions, which appeared to be contingent on their respective circumstances (e.g., stage in career). While two young players focused on the time to improve, one of the older players expressed disappointment with the postponement. Moreover, while the players were grateful that the OGs were held, some expressed ethical reservations, as exemplified by Fredrik:

“We’ve been allowed to play sports while they [Japanese citizens] have not been allowed to go to cafes. They aren’t allowed to that. And then we are standing there fighting over a ball, which in the grand scheme of things does not matter when there’s been a pandemic.”

#### 3.2.2 Uncertainty during the pandemic Olympic Games

The players unanimously viewed the OGs as unique due to their quadrennial occurrence, leading to fewer medal opportunities than biennial ECs and WCs. This rarity amplified the significance and pressure, as echoed by former participants. Dina highlighted the remarkable simplicity with which distractions can ensue, while Fredrik pointed to the increased investment in their performance, resulting in more people depending on them. The colossal scale of the OGs and the heightened media attention further differentiate it from other championships. Overall, this creates a high-pressure environment filled with new stimuli and impressions that can inspire and interfere with one’s focus. In Bea’s words:

“There are many factors that are a bit more disturbing because … when it’s the OGs, you have thousands of athletes from different nations, you have men’s handball teams, you have big stars walking around in the Olympic Village, which—the Olympic Village is a lot bigger, you usually live in a hotel, and then you go down to the first floor and eat breakfast, and then you go back up to the room again. While there, you are supposed to walk maybe from [laughter] 500 to 1500 meters to get to the dining hall, so there’s a lot more, yeah, other factors.”

The logistics are more complicated than in other championships, and staying in the Village comprises a variety of potential disruptions. Some remarked that simply arriving was “completely overwhelming.” According to Blumenstein and Lidor (2008), there is a “unique psychological and social atmosphere in the Village that is felt by even the most experienced athletes and coaches” (p. 288). This aligns with other literature—the OG context distinguishes itself from other championships (e.g., Arnold and Sarkar, 2015; Blumenstein and Lidor, 2008; Gould et al., 1999; McCann, 2008). The first-time participants particularly emphasized all the new impressions. Dina remarked that it is an entirely different bubble compared to other championships and that there is much to learn about what the OGs entail.

“I’ve always thought that the first OGs really were the biggest because it’s the first time you experience it and you do not know what you are heading into … You have a dining hall that’s the size of three football fields, and there’s a fitness center that is just gigantic where it’s like maybe 50 treadmills and there are 50 bikes, and there are a bunch of squat racks … but then ahead of the next OGs again it’s sort of the same as it was in the first one.” (Bea).

The Tokyo OGs further differentiated themselves due to COVID-19 restrictions. There were strict regulations, including daily testing, mask mandates (also during training), and not being allowed to leave the Olympic Village. The players embraced the situation in distinct ways. Bea focused on adaptability, implying there was no point in being irritated by factors beyond their control. Although he was grateful for the experience, Fredrik felt that the restrictions partly ruined his Olympic experience—for instance, being unable to walk around more freely. Anna illustrated the overall situation as follows:

“Everything was rigorous then, a lot of routines on mask-wearing, who we were supposed to be with. We were very cautious with everyone else we were with because we did not know—for example, we suddenly heard that there had been [COVID-19] in different apartments. Yeah, it was sort of uncertain, and we tried to kind of be strict at the same time as we were in the dining hall with everyone else, and it was kind of—you were also around others all the time, but I do think in a way that it was defined by the fact that it was [COVID-19] there.”

Despite pandemic restrictions, OG athletes resided in the Olympic Village. The male players valued the social life at the Tokyo OGs, where they could exchange experiences with other Norwegian athletes. Living in close quarters could also be demanding, with shared rooms and 6–10 players per apartment leading to limited privacy. Staying in the Olympic Village offers many internal and external stressors (e.g., Arnold and Sarkar, 2015; Blumenstein and Lidor, 2008; Kidd, 2013; Kristiansen et al., 2013). The lack of privacy, the distractions, and the general stressful atmosphere are among the psychological barriers that can interfere with preparation (Blumenstein and Lidor, 2008, p. 288). Fredrik pointed out the impact of this constant proximity:

“I’m usually pretty good at sort of resetting and dealing with the things that I cannot do anything about, but that’s easier when you have the opportunity to disconnect and when you aren’t living on top of someone for six weeks … During the fourth, fifth week it got a bit more challenging [laughter] than it was during the first days maybe.”

#### 3.2.3 Rebounding from setback: post-Olympic reflections

Neither team reached their goal during the OGs. Most women’s team players had gold medals from ECs and WCs, and their collections were missing Olympic gold. Being part of a team with a genuine chance of winning, combined with the marginal semifinal loss during the Rio OGs, generated immense pressure and a notion of it being all-or-nothing. As Bea said, “It’s almost like you are sort of more scared to lose than you are happy to win.”

“In 2016, we lost the OGs on the smallest margin in the semifinals … I think there was sort of a lot surrounding that year, and it was like “Okay, now it’s four years and then it’s gonna be us” … And then an extra year went by as well with [COVID-19], and it could actually have been good for us, we got some players back … But then things still got a bit like, yeah, just like, “Okay, we have to.” We have to so badly, and then we get exactly the same team we lost against in ‘16 in exactly the same semifinal. It’s like so much that influences things.” (Dina).

The women’s team ended with a bronze. Bea talked about the team’s experience dealing with the semifinal loss and how they refocused for the last game. She described individual variations in coping, some needing more time than others. She said, “You give room so that we can all be disappointed today, but when we get up tomorrow and are playing the bronze final, then people need to be ready.” While they were satisfied with turning around their performance, they were frustrated not to get the opportunity to fight for the gold.

“It’s clear that we were enormously disappointed after the OGs because we had such high expectations … But at the same time I was very, very proud of that bronze medal, and it meant so much to me to get an Olympic medal even if it wasn’t gold.” (Caroline).

Irrespective of their disappointment, the women agreed that their team had prepared well—in fact, Dina felt they were more prepared than at numerous prior championships. Overall, they reflected on how several players were overly invested, with many focusing on this being their last chance. Dina suggested that they could have talked more about the personal significance they placed on the OGs, as doing so could have helped them work their way out of it together. Preparing even more systematically at the team level (see, e.g., Collins and Cruickshank, 2015; Gould and Maynard, 2009) was something several players mentioned. The female team went on to win the OGs gold medal in Paris 2024.

The men’s team wanted to fight for medals but lost two out of five games only to be put up against Denmark (reigning world champions) in the quarterfinal. They attempted it wholeheartedly but seemingly “ran out of wind.” Their post-OG reflections varied significantly. Georg was satisfied with their COVID-19 preparations, while Endre valued the experience despite feeling the efforts were fruitless. He had to lower his expectations in Tokyo, and mental exercises became crucial in coping. Using his SPC for emotional support, he received assignments to focus on outside handball, such as making playlists and finding control in the “Olympic life”:

“[My mental coach] helped me through those periods and especially considering the situation we were in, where we were locked so to speak inside a barrack that the whole world thinks is so incredibly great and exciting, and it’s an apartment of 50 square meters with paper-thin walls and you live [with] six people inside it or seven.”

Fredrik was content with his preparations but felt mentally worn out after a demanding season. He acknowledged that even though they had a good idea of what to expect as first-timers, some lessons can only be learned through experience. The men’s team lost the quarterfinal again in the 2024 Paris OGs after beating, for instance, the reigning Olympic champions (France) in the group stage to no avail. Fredrik gets the concluding remarks, reflecting on the OGs retrospectively:

“I thought it was very, very tough. I’d just played, I do not know 60 to 70 games with [club] and the national team, and then I had 10 days of vacation before it was [time for] pre-camp and then you were going to Tokyo, and I’m actually exhausted after a long season, and suddenly you are put in a situation where you are supposed to perform now. So, it was clear that you were not at 100% neither physically nor mentally when you were down there in Tokyo, but, I do not really think the other nations were either.”

#### 3.2.4 Balancing life and sports in a pandemic—recapitulation

Research highlights the contrast between the structured environment of elite athletes and the aimless circumstances brought on by isolation, which result in the loss of motivation and in psychological distress (Gupta and McCarthy, 2021). The novel situation (Lazarus and Folkman, 1984; Thatcher and Day, 2008) of a pandemic created ambiguity and complicated athletes’ coping efforts. Uncertainty about lockdowns and lack of control have also been reported as demanding elsewhere (Whitcomb-Khan et al., 2021). Changes in daily life, including isolation from friends, teammates, and support staff, and the importance of social support during the pandemic are also significant (Gupta and McCarthy, 2021; Rogers and Werthner, 2023). The impact of these changes is hard to determine fully but likely varies individually and affected athletes’ well-being and performance levels (Schinke et al., 2020). Again, this was supported by the sport psychology professionals in Lundqvist et al.’s study (2022).

Generally, the appraisals of the pandemic were contingent on the players’ contexts as disparities emerged (e.g., financial status and time in one’s career). Postponing the OGs meant deferring retirement for some while providing time to enhance performance for others. Similarly, the players’ perceptions in this study seemed to be impacted by their circumstances. Coping efforts were also dependent on the individual: While some sought emotional support and reappraised the situation to gain perspective (e.g., emotion-focused coping; Lazarus and Folkman, 1987; Nicholls and Polman, 2007), others were quicker to accept that there was nothing they could do about the situation (similar to a mindful/acceptance approach; see, e.g., Gardner and Moore, 2017).

## 4 General discussion

The current study qualitatively explored how seven Norwegian handball players with different experience levels prepared for and coped with the Tokyo OGs. The results revealed extensive preparations involving numerous areas (e.g., mental, physical, tactical, and practical). These multidisciplinary preparations align with those reported in previous studies (Arnold and Sarkar, 2015; Collins and Cruickshank, 2015; Gould and Maynard, 2009). The indications of differences between the teams and individuals signify that there are several ways to prepare for the OGs. The pandemic caused an unprecedented situation that affected athletes worldwide—including the present athletes. Lockdowns and restrictions altered training practices (e.g., Bowes et al., 2020; Clemente-Suárez et al., 2020; Gupta and McCarthy, 2021; Whitcomb-Khan et al., 2021), and athletes experienced several negative consequences—including loss of motivation (Gupta and McCarthy, 2021; Oblinger-Peters and Krenn, 2020; Whitcomb-Khan et al., 2021), financial concerns (Bowes et al., 2020; Schinke et al., 2020), an increase in stressors and stress levels (di Fronso et al., 2022; Reardon et al., 2021; Schinke et al., 2020, p. 270), and a higher prevalence of symptoms of mental health issues (Pensgaard et al., 2021). Nevertheless, “Moments of quietness present openings to reflect, re-evaluate, revise, and reform plans” (Schinke et al., 2020). In a recent review, Kuo et al. (2025) investigated how the COVID-19 pandemic influenced elite athletes’ mental health symptoms, especially focusing on sex differences. The outcome showed that female athletes experienced higher levels of anxiety, depression, and distress compared to male athletes, highlighting the need for targeted mental health resources.

Moreover, the pandemic provided opportunities to explore non-athletic identities (e.g., Reardon et al., 2021; Wadsworth and Hargreaves, 2021) and broadening perspectives—as is evident in the current sample. Studies suggest that despite the challenges posed by the pandemic and the OGs postponement (e.g., quarantines, social isolation, loss of sporting activities and motivation), the circumstances were, at some point, accepted and positively reframed (e.g., a chance to rest, mend injuries, and improve skills; Gupta and McCarthy, 2021; Oblinger-Peters and Krenn, 2020; Rogers and Werthner, 2023; Whitcomb-Khan et al., 2021). The players in our study handled the unavoidable delay of the OGs with perspective, though individual impacts varied based on circumstances. While players generally stayed positive, some found the situation mentally draining, possibly impacting preparations and performance.

### 4.1 Limitations

This study illuminates the realm of Olympic preparations among elite Norwegian athletes, an area that has remained largely unexplored. It offers valuable insights and broadens understanding of how athletes’ approach and manage training for global events with added challenges. However, it is essential to acknowledge that despite these advancements, certain limitations within this research persist. Despite thorough pilot testing, the first author’s novice interviewing status might have influenced the process. The two co-authors helped overcome these challenges —including planning, the execution of data collection, and analyses. Nonetheless, we openly acknowledge this, striving for transparency by detailing our roles as researchers, our interests, and the reasoning behind our methodological choices.

The elite standing of the participants served as both a strength—offering a rare and information-rich sample—and a challenge due to time limitations and anonymity requirements. The elite status also resulted in a limited sample size, resulting in some themes being less explored. Positional demands are therefore promising areas for future sports psychology research. In a purposefully sampled group of elite athletes, small sample sizes can pose limitations, as dominant voices may disproportionately influence the findings—potentially skewing the interpretation of interviews and overshadowing less vocal perspectives. Including participants with diverse backgrounds, as well as both experienced and less experienced athletes, can help ensure a richer, more nuanced and more balanced insights. Nevertheless, given the study’s specific aim, a smaller, particular sample was necessary. The narrow focus of the interviews, the rich information provided by experienced athletes with unique insights gave the study information power (Malterud et al., 2015) despite having a smaller, but with an exclusive group of Olympic participants.

Mikecz (2012) noted a potential limitation with research on elites—they may be conditioned to represent themselves and their organization. Accustomed to expressing their views, they might lean toward monologues rather than addressing challenging questions. The first author encountered this in one interview—but with ample time to probe the answers thoroughly, this occurrence caused no challenge. We aimed for more member reflections, but the elite players’ time constraints made this tricky. To address this, a peer of the first author helped decipher unclear passages, while the co-authors assisted in the interpretation.

The use of online interviews enabled us to conducted the interviews despite covid restrictions athletes traveling extensively. The interview situation might hinder reading body language or creating a comfortable setting, therefore potentially reducing the ability to build rapport (Seitz, 2016). With this in mind, athletes are used to communicating with family and friends due to hectic travel schedules. Furthermore, research examining the use of Zoom in qualitative data collection, Archibald et al. (2019) discovered that the majority of participants (69%) preferred it over in-person interviews, phone, or other videoconferencing platforms; labeled as “the closest thing” to a face-to-face meeting (pp. 3–4). Thus, using a videoconferencing platform for the current study was considered a sifficent method.

Finally, this retrospective study may be subject to recall bias, as the players might have forgotten specific details over the Olympic cycle. The players’ recollections might also have been influenced by their performance. The interviews were conducted from November 2021 to January 2022, not long after the Tokyo OGs—thus, recall should not have been a significant issue. While prospective methods are recommended (Gould and Maynard, 2009), these were not achievable for this study—due to the abovementioned restrictions.

### 4.2 Future research and practical implications

Ideally, research could track athletes across an Olympic cycle, combining prospective and retrospective interviews with time-based observations, including support personnel’s perspectives. Where time and resource constraints and participant recruitment challenges allow, future studies should use prospective and retrospective methods when feasible. Notably, interviewing athletes from different sports may have provided different types of preparation with a more extended preparation phase and, therefore, the opportunity to focus more on training for the OGs and how athletes dealt with the pandemic.

The Olympic atmosphere, with its distractions, limited privacy, and increased pressure, can overwhelm first-time participants. Consequently, SPCs may need to adjust their strategies (Arnold and Sarkar, 2015; Blumenstein and Lidor, 2008), and those coaching aspiring Olympians should underline the unique nature of the OGs compared to other competitions.

The interviews suggest that both teams were well-prepared with their unique traditions. The women’s team, with a more established culture (Hemmestad and Jones, 2019), seemed to have had more time to develop their traditions, potentially benefiting their preparations. Paraphrasing two OG SPCs reflections (Pensgaard and Abrahamsen, 2012), their experiences highlight both advantages and drawbacks of short-term engagements pre-Olympics with a team, whereas long-term involvement predominantly demonstrates positive outcomes.

Research has shown that first-time Olympians who generally feel well-prepared might still be caught off guard and unprepared for the event’s magnitude (Jensen et al., 2014). Still, preparing and discussing potential “what-ifs” is recommended for teams going into the OGs and patiently nurturing the necessary individual and team coping skills over time.

## Data Availability

Due to confidentiality concerns, full interview transcripts cannot be shared. However, anonymized excerpts that support the themes identified in the study are available from the corresponding author (henrik.gustafsson@kau.se) upon request.
